# Asthma 101 for Schools: Successes and Challenges in Transitioning to Online Delivery

**DOI:** 10.3389/fpubh.2016.00011

**Published:** 2016-02-01

**Authors:** Alexandra Catherine Hayes Nowakowski, Henry Joseph Carretta, Julie Kurlfink Dudley, Jamie R. Forrest

**Affiliations:** ^1^Behavioral Sciences and Social Medicine, Florida State University College of Medicine, Tallahassee, FL, USA; ^2^Florida Department of Health, Tallahassee, FL, USA

**Keywords:** asthma, schools, online training, program evaluation, Florida

## Abstract

Florida Asthma Program staff worked with evaluators from the Florida State University College of Medicine to assess participation and quality of the American Lung Association’s Asthma 101 asthma management education program for school faculty and staff between 2011 and 2014. This included transitioning the program to an online training format for the 2013–2014 school year. Asthma 101 helps school personnel master the basics of asthma physiology and management, with content tailored specifically for elementary and secondary educational settings. The program is assessed with questionnaires at multiple timepoints, yielding a quasi-experimental evaluation design. Evaluators reviewed quantitative data from pretests and qualitative and quantitative data from post-program satisfaction questionnaires. Program spreadsheets listing the dates for delivery and number of attendees were also reviewed. Overall, evaluation findings were positive. In the 2011–2012 program year, 16 different course sessions were offered, and more than half of enrolled participants came from Title I schools. A total of 228 people were trained. In the 2012–2013 program year, 19 different course sessions were offered. Enrollment totals (638) and matching pre- and posttest totals (562) soundly exceeded the target metric of 425. At least 170 (27%) of a total of 638 participants could be verified as coming from the target demographic of Title I school faculty and staff. In the 2013–2014 program year, the course was offered online on a rolling basis *via* the Florida TRAIN course management system. Enrollment remained high and learner outcomes remained consistently strong across all content areas for knowledge and satisfaction. A total of 406 people participated in the training; complete pre- and posttest data were available for 341 of these individuals; and satisfaction data were available for 325. Of the 406 trainees, 199 (49%) reported working for Title I schools. Evaluation yielded very positive results. An overwhelming majority of participants reported finding the course consistently strong across the board and highly impactful for their own ability to help students manage their asthma effectively. Most participants also reported that they would change/improve their asthma management behaviors in the workplace. Recommendations were developed to help expand future program reach.

## Introduction

Evaluation and implementation of the Asthma 101 courses discussed herein occurred under a Cooperative Agreement between the Centers for Disease Control and Prevention and the Asthma Program and its staff at the Florida Department of Health. Asthma 101 is a 1-h course developed by the American Lung Association that teaches school personnel how to help students with asthma. The course covers basic asthma anatomy and physiology, signs and symptoms of respiratory distress, when and how to give medications, Asthma Action Plans, how to use asthma management equipment, how to make environmental modifications to control triggers, and other related content.

In the United States, the potential for impacting asthma management *via* educational contact with school personnel is high. Teachers and staff are not necessarily involved in direct delivery of care the way a school nurse or health liaison might be but are generally the people present when a student with asthma experiences an adverse event. Thus, Asthma 101 focuses extensively on preparing teachers and staff to respond quickly, confidently, and effectively to asthma events in the student population. The course also prepares school personnel to talk with students about their needs related to asthma management and to link them with services and resources that can help them get the most out of each school day and achieve better quality of life.

The overall goal of the evaluation was to see how well the Asthma 101 training was delivered in Florida for the 2011–2012, 2012–2013, and 2013–2014 program years. Evaluators used the following questions to assess program outcomes:

1. How many people participated in Asthma 101 sessions?
2. How many participants were from the target audience?
3. How satisfied were participants with the quality of the Asthma 101 program?

From this preliminary evaluation of Asthma 101 comparing the in-person and online versions of courses for public school audiences in Florida, we aimed to develop recommendations for improving the training and its delivery in the future. To that end, the Asthma Program continues to share and implement evaluation findings with key stakeholders including American Lung Association staff, Florida Asthma Coalition School Workgroup members, local asthma coalitions, and the Centers for Disease Control. Findings are presently being used to improve course marketing and delivery in the new Centers for Disease Control funding cycle for 2014–2019. In the current project period, Asthma 101 is one of the several continuing education courses that partners in the education sector can take to achieve Asthma Friendly School recognition.

### Background

Published studies of Asthma 101’s effectiveness demonstrate that the course has consistently and significantly improved asthma management knowledge and skills among education professionals (1). However, no studies have yet explored the impact of this course when delivered in a Web-based format. Consequently, no studies have yet compared results from an online version of the training to those from an in-person one. Thus, we present our work as a preliminary effort to bridge this gap and encourage more in-depth activity in the future, using both Asthma 101 and other asthma management training curricula.

Substantially more literature addresses the short-term impacts and long-term value of asthma management education programs that engage professionals who work with children. Zahran et al. (2) stress that the most effective asthma management education programs target a variety of people who influence children’s daily lives and behavior, including teachers. School-based programs can reduce barriers to effective asthma control, promote proper use of medications and devices, and promote sustainable behavior change (2).

A growing literature also addresses the potential of computer-based asthma management education to promote population health and increase care capacity. Cicutto et al. (3) found that a combination of in-person training, clinical support tools, patient education materials, and spirometry assessment significantly improved provider confidence and care quality when delivered with Web-based components. Early computerized self-management interventions aimed at children also performed well, with participants showing better knowledge and behavior as well as lower health-care utilization (4). Subsequent efforts (5–7) have likewise demonstrated that computer-based trainings can improve asthma management outcomes for children and families in several key domains across a variety of social and economic locations.

More contemporary trainings have focused specifically on Web-based delivery of content, with similarly strong results. Specific positive impacts include consistent use of controller medications, reduced use of reliever medications, and fewer urgent physician visits (8). As with the older computer-based trainings above, most of these trainings have engaged children and their families, but not other people with strong influence in children’s daily activities, such as schoolwork and active play. No other evaluations of online asthma management trainings that directly engage educators have yet been published in peer-reviewed journals.

Thus, we sought to describe and compare the impacts of the American Lung Association’s highly successful Asthma 101 course when delivered in both in-person and online formats for educators in Florida. Building on evidence from published literature suggesting that asthma management education can yield especially impactful results in low-resource areas (9, 10), we focused our efforts on enrolling teachers from Title I schools whenever possible. Title I schools are located in low-income geographic areas, and enrollment from medically underserved students is often high. Likewise, students living in areas served by Title I schools are likely to get exposed to asthma triggers more frequently than their peers in more affluent areas. Thus, working with Title I schools as a focus offers strong potential for near-term impact with asthma management interventions.

## Materials and Methods

Enrollment in Asthma 101 was open to education professionals employed at public schools throughout Florida. The training was marketed to both Title I and non-Title I schools. Target enrollment numbers for overall and Title I participation are detailed in Section “Results,” along with metrics about the extent to which each target was achieved.

Content elements for the in-person and online versions of the Asthma 101 training were identical. To put the course into the Florida TRAIN system, Department of Health program staff used the same slides that were given to presenters for the face-to-face courses. Both the in-person and online versions of the training lasted for approximately 1 h. All versions and iterations of the training during the three program years included in the data stream were evaluated with comparable strategies as described below.

Participants in the 2011–2012 course sessions took a short pre-questionnaire that measured their knowledge at baseline and a post-questionnaire that measured their satisfaction with the course. Participants in the 2012–2013 course sessions took a pretest that measured their knowledge at baseline and a posttest that measured both their knowledge (of the same items measured on the pretest) and satisfaction with the course. Participants in the 2013–2014 online course took a pretest that measured their knowledge at baseline, a posttest that measured their knowledge of the same items, and a satisfaction survey for the course as a whole.

ALA program tracking spreadsheets, attendance logs, pretests, and posttests were shared with evaluators by the ALA staff. Tracking spreadsheets provided details on participation by school, as well as demographic information about schools themselves. These spreadsheets were used by the evaluators to record how many students actually completed the course after review of attendance logs and questionnaires. These data were recoded in Excel to reflect level of agreement for questions about course quality. Univariate statistics were computed using Excel for all items of interest. To assess knowledge gain, scores on the pre- and posttests were compared using paired *T*-tests for each knowledge question.

A follow-up survey was developed to measure knowledge gain, but it was not implemented prior to the end of Grant year 3. These data were also excluded from the Grant year 4 and Grant year 5 evaluations for purposes of consistency. In the new online training format, trainees take three questionnaires: one at baseline, one immediately after taking the course, and one 2 months after taking the course. All three surveys measure knowledge on the same 10 items, and the post-questionnaire continues to look at the course quality. However, fewer than 20 of the 369 trainees with complete pre- and posttest data actually took the follow-up survey, likely because doing so was not required to receive course credit.

This evaluation was approved by the Florida State University Human Subjects Committee. Initial approval was granted in Spring 2012, with annual renewal through the close of the project period in Fall 2014. All activities for the evaluation were conducted in accordance with requirements for human subjects research outlined in the United States Code of Federal Regulations, Chapter 45, Part 46. To protect participant confidentiality, all data were de-identified prior to the entry into central databases. We report evaluation findings only in aggregate and disclose no protected health information about specific participants at any point during reporting of results.

## Results

### Tracking Spreadsheet (Recruitment/Participation)

A total of 228 people took the Asthma 101 training between December 2, 2011 and August 31, 2012; matched pretests and post-surveys were available for 203 of these participants. In the 2012–2013 program year, 638 people took the course and 562 had matching tests. In the 2013–2014 program year, 437 people took the training and 369 had matching tests. Training enrollment targets were exceeded for 2012–2013 and 2013–2014 and were considered met for 2011–2012 given the compressed timetable for project activity. On average, over 425 people took the course every year, surpassing the average target metric of 400 established at the beginning of the program period.

A total of 16 Asthma 101 course sessions were offered in 2011–2012. Of these, 10 sessions were offered at Title I schools in either Duval or Miami-Dade County. No sessions were offered at Title I schools in Orange County during the 2011–2012 school year. At least 110 out of 228 (48%) total participants for 2011–2012 reported working in Title I schools. This number is likely higher because not everyone reported whether or not they work in a Title I School.

A total of 19 Asthma 101 course sessions were offered in 2012–2013. Of the 638 total participants, 428 (67%) came from schools in Duval County. Other trainees came from Broward, Leon, and Santa Rosa counties. Only 17 (3%) of the 638 participants had previously taken an Asthma 101 course. At least 170 (27%) of the 638 total participants came from Title I schools. This is a conservative estimate because several trainings were delivered to mixed school audiences, and thus some enrollees could not be verified as Title I even if they actually worked at Title I schools. Among the 562 participants with matching pre- and posttests, 151 (27%) could be verified as working in Title I schools.

In 2013–2014, the course was offered online *via* Florida TRAIN. Of the 406 total participants, 382 people answered the question about Title I status and 199 (52%) of these individuals reported working in Title I schools. Overall, 49% of the total course participants could be verified as working in Title I schools. This brought us close to our goal of having about 60% of Asthma 101 participants drawn from Title I schools. County of origin and previous experience with Asthma 101 were not assessed by the online course management system.

#### Knowledge Assessment

In all three program years, assessment of knowledge at baseline revealed that participants entered the course with a wide variety of asthma knowledge. Participants scored highly on some measures of knowledge while not appearing very knowledgeable on others. Correct response rates for asthma knowledge questions ranged from a low of 32% to a high of over 98% on pretest. Results for specific knowledge assessment questions at baseline are shown in Table 1.

**Table 1:** **Knowledge metrics on pretest**.

Question	Percent of responses correct (2011–2012)	Percent of responses correct (2012–2013)	Percent of responses correct (2013–2014)
There is no cure for asthma	80.8	84.9	90.3
Although asthma causes breathing problems, asthma episodes are not dangerous	93.8	96.4	97.9
Taking medications every day can help prevent an asthma episode	–	87.9	86.5
A person should take steroids for asthma to grow muscle mass for better athletic ability	86.8	89.7	91.2
Inhaled steroids have the same side effects as steroids taken by mouth	55.0	68.5	66.8
Very little can be done to reduce environmental triggers of asthma	69.0	76.1	78.2
Asthma medications are not addictive	51.4	55.9	66.6
People with asthma should not participate in sports	97.2	96.4	98.2
Asthma episodes always occur suddenly, without warning	71.5	78.1	84.4
Asthma can be caused by emotional stress or other psychological problems	31.9	82.0	83.6
A rescue or reliever medication (albuterol) helps during an asthma episode by making you breathe faster	47.6	63.3	65.1

Participants in programs delivered during the 2011–2012 year appear to have come in with strong knowledge about the dangers of asthma attacks, as well as the basic physiology of asthma and what kinds of activities people who have asthma can do. Baseline knowledge of asthma management medications and environmental triggers was much more limited. Participants in Asthma 101 during the 2012–2013 year appear to have started the training with strong knowledge about the dangers of asthma attacks, as well as the basic physiology of asthma and what kinds of activities people who have asthma can do. Baseline knowledge of asthma management medications, warning signs for attacks, and environmental triggers was more limited. Participants for the 2013–2014 year began with strong knowledge about the dangers of asthma attacks, as well as the basic physiology of asthma and what kinds of activities people who have asthma can do. Baseline knowledge of asthma management medications and environmental triggers was more limited.

In 2012–2013 and 2013–2014 only, participants were asked the same set of knowledge questions after taking the course. Results are summarized below and shown in Table 2. The third question was only included on the pretests for 2012–2013 and 2013–2014.

**Table 2:** **Knowledge change from pretest to posttest**.

Question	Percent of responses correct (2012–2013)	Change from pretest to posttest (2012–2014) (%)	Percent of responses correct (2013–2014)	Change from pretest to posttest (2012–2014) (%)	Overall impact of training
There is no cure for asthma	91.1	+6.2***	95.9	+5.6***	Significant improvement
Although asthma causes breathing problems, asthma episodes are not dangerous	97.5	+1.1	97.9	+0.3	No consistent change
Taking medications every day can help prevent an asthma episode	87.9	+11.2***	93.2	+6.7***	Significant improvement
A person should take steroids for asthma to grow muscle mass for better athletic ability	91.1	+1.4	89.7	−1.5	No consistent change
Inhaled steroids have the same side effects as steroids taken by mouth	71.4	+2.9	75.3	+8.5**	No consistent change
Very little can be done to reduce environmental triggers of asthma	70.1	−6.1	87.8	+9.4***	No consistent change
Asthma medications are not addictive	70.6	+14.8***	78.9	+12.3***	Significant improvement
People with asthma should not participate in sports	97.8	+1.4^†^	99.4	+1.1^†^	Marginal improvement
Asthma episodes always occur suddenly, without warning	81.9	+3.7*	92.3	+7.9***	Significant improvement
Asthma can be caused by emotional stress or other psychological problems	88.3	+6.2***	75.4	−8.2*	No consistent change
A rescue or reliever medication (albuterol) helps during an asthma episode by making you breathe faster	66.2	+2.9	70.7	+5.6*	No consistent change

*^†^*p* < 0.10*.

*p < 0.05.

**p < 0.01.

***p < 0.001.

The Asthma 101 course produced significant improvement on four of 11 knowledge items, marginal improvement on 1 item, and no consistent change on 6 others. In some cases, this owed mainly to high levels of baseline knowledge at pretest. Knowledge appeared to remain relatively low on questions about medications and environmental trigger management even after the training. This may recommend improvements to the Asthma 101 training materials and/or delivery in future program years as the online format is refined. Likewise, question wording may have contributed to persistence of low levels of knowledge based on pre- and posttest results. For example, some of the items contain “double-barreled” or ambiguous wording that may have confused participants even if they did gain knowledge about those general content areas from taking the course.

#### Satisfaction Assessment

Participants were asked to rate the quality of specific elements of the training. A large majority of participants found the training excellent across the board. One set of questions asked how satisfied participants were with key course elements. Participants consistently reported high levels of satisfaction with all elements of the course, and nearly all who did not report the highest level of satisfaction reported being “somewhat satisfied.” Results for specific course content elements are shown in Table 3.

**Table 3:** **Satisfaction with Asthma 101 course elements**.

Question	Very or somewhat satisfied (2011–2012)	Very or somewhat satisfied (2012–2013)	Very or somewhat satisfied (2013–2014)
… the amount of information?	134 (97%)	477 (85%)	312 (96%)
… the quality of the information?	134 (97%)	476 (85%)	307 (94%)
… the organization of the program?	134 (97%)	475 (85%)	291 (90%)
… the relevance of this program to their work?	135 (98%)	477 (85%)	304 (94%)
… the written materials provided?	133 (96%)	454 (82%)	308 (95%)
… the program overall?	134 (97%)	457 (81%)	271 (83%)

A second set of quality questions asked participants to rate their agreement that the Asthma 101 program improved their knowledge and preparedness for future asthma management efforts. A large majority of participants felt strongly that the Asthma 101 program was useful for improving their asthma management knowledge (between 80 and 94% for in-person trainings, 79% for online training) and preparedness (between 84 and 94% for in-person trainings, 80% for online training). Of those who did not report “strongly agreeing” that the program was useful in these two areas, nearly all participants “moderately agreed” for both the in-person and online formats.

Participants were also asked how likely they would be to use what they learned at work. School personnel who took the Asthma 101 course face-to-face were almost uniformly “very likely” to use what they had learned at work, with the majority of remaining participants reporting that they were “somewhat likely” to implement their knowledge in the workplace. For the first year of in-person training, 93% of participants chose these response options; in the second year, this proportion dropped but remained high at 82%. Results for the online training were near the upper bound of those for the in-person course, with 89% of participants reporting that they were “very likely” or “somewhat likely” to implement their new knowledge. We note that these measures only captured participants’ intent to change their behavior, not action taken or the maintenance thereof across time.

Participants were asked which parts of the program were most useful for them. Results are shown below in Table 4 and are presented as raw counts due to the “select multiple” structure of this survey question. Participants in Asthma 101 for all three program years reported finding the content on signs and symptoms of respiratory distress most useful, followed closely by content on Asthma Action Plans. Large proportions of respondents felt that each named aspect of the course was useful.

**Table 4:** **Perceived utility of Asthma 101 course content items**.

Program component	Number reporting as most useful (2011–2012)	Number reporting as most useful (2012–2013)	Number reporting as most useful (2013–2014)
What is asthma?	59	430	125
Signs and symptoms of respiratory distress	86	467	213
When to give medications	63	435	114
Asthma Action Plan	83	454	150
How to use equipment	53	417	104
How to make environmental modifications	54	421	138
Other	6	0	30

Participants were also given the opportunity to recommend improvements to the training course. Four key themes emerged across program years. First, participants wanted more outreach and relationship building with their schools. Second, more information about asthma triggers was desired. Third, participants wished for a more concise and convenient course format. Fourth, more video and other media examples were strongly recommended.

## Discussion

Evaluating the Asthma 101 training for in-person and online delivery formats revealed strong performance for the course overall and comparable performance for the two versions. We also found substantial evidence to suggest that offering Asthma 101 online improved reach among diverse populations throughout Florida and increased potential for enrolling our highest priority target trainees – i.e., teachers in Title I schools. We used these results to develop recommendations for future activity involving Asthma 101 in Florida. In the process, we reflected on key strengths and limitations of this evaluation framework, as well as the two versions of Asthma 101 that we evaluated.

Our approach to evaluating Asthma 101 had a number of notable strengths. First, we only analyzed data from respondents who actually contributed those values themselves, rather than attempting to impute any missing data. Second, we analyzed data from a variety of different school districts with diverse geographic locations and density of different types of resources that may promote effective asthma management. Thus, we feel reasonably confident that Asthma 101 has strong potential for positive impact when delivered face-to-face and/or online in diverse Florida communities.

Our evaluation also had a number of important limitations. First, we could not compare data directly for in-person and online versions of the training within a single program year. Second, we also had only 1 year of data for the online version as opposed to 2 years for the in-person training. This evaluation is best viewed as an early-stage effort focused chiefly on user perceptions and satisfaction, with some very basic data elements capturing knowledge gain and likely behavior change. The literature demonstrates that intent to act can serve as a good predictor of future behavior but is best viewed as the sign of an intervention’s potential rather than its long-term impact. Likewise, we provide information about challenges with transitioning to the online version to offer some contextualization for making predictions about how the online training will perform in the future. As noted above, we also cannot generalize our findings from evaluating Asthma 101 in Florida to make projections about its utility for other states or how online versus in-person versions of the trainings would compare to one another in other geographic locations.

The Asthma 101 training itself also has important strengths and limitations, both within and across versions. The training performed strongly in both in-person and online formats at improving knowledge – a key strength. Participants also consistently reported high likelihood of using what they learned in the training; these results were again comparable for both training formats. However, we had no way of knowing how much those intentions actually translated into concrete behavior change for either version of the training. Likewise, because both versions of the course were designed with only a single assessment of knowledge and satisfaction post-course, we could not examine either retention of information or perceived utility of that information across time. These limitations represent opportunities for improving the Asthma 101 training materials – and resultant evaluation capacity – in future years for both in-person and online versions of the course. As other asthma management evaluation and implementation professionals look toward building on the recommendations we offer for future work, we strongly encourage close attention to differentiating intent from action in people who have taken Asthma 101.

## Conclusion

Results from evaluation of the Asthma 101 course for Grant years 3, 4, and 5 showed strong performance for both in-person and online formats of the training, potential for increased reach, and impact *via* leveraging of the Web training platform. Based on these results, our evaluation team recommended three key improvements to the program’s planning, content, and delivery in Florida that are now being implemented in the new grant cycle. Specifically, we recommended that the Asthma Program (1) continue reaching out actively to Title I schools, (2) promote the online version of Asthma 101, and (3) revise strategies for implementing the follow-up survey across course formats. These recommendations build on cumulative findings from 3 years of evaluating Asthma 101 in Florida, within the context of a successful but challenging transition to online course delivery during Grant year 5.

### Continue Reaching Out Actively to Title I Schools

The Florida Asthma Program and Coalition have an opportunity to reach faculty and staff who work with low-income children more easily using the online training format, which enables teachers to take trainings during the summer, outside of school hours, or during free periods. Results from the first year of online training suggest that the TRAIN system does indeed make it easier for Title I school personnel to connect with and participate in the Asthma 101 course, with enrollment of Title I personnel nearly doubling for the 2013–2014 program year.

### Communicate the Advantages of the Online Course Available through Florida TRAIN

Results from previous years’ evaluations indicated strong demand for an online course format and high enrollment in the first year of online training corroborated interest in the Web-based course. The online course minimizes time costs of training and fits with participants’ schedules, offering a variety of advantages over the traditional in-person format. As noted above, this appears to have been particularly helpful for Title I school personnel, a key target demographic for the training.

### Revise Strategies for Implementing the Follow-up Survey

The biggest shortcoming of the online training in its first year was its failure to collect follow-up data for the vast majority of participants. Asthma Program staff implemented previous years’ recommendations for collecting data on behavior change *via* this follow-up survey, but because only a very small number of people responded to the questionnaire, the data were not substantively useful. This could be resolved in future iterations of the training by linking follow-up survey completion to additional credit or credentialing. For example, participants with professional licenses or certifications – which accounts for a large majority of public school teachers – could take a short refresher module and then complete the follow-up survey for an additional continuing education credit.

We are also focusing more for the 2014–2019 project period on contact and participation, given positive results from the initial evaluation of satisfaction and perceived impact, as well as the strong evidence base for the effectiveness of Asthma 101 in other states. As mentioned previously, Asthma 101 is currently one of the several asthma management education courses that partner schools in Florida can offer if they wish to achieve Asthma Friendly School recognition from the FAP. This recognition is an official credential that can support schools in their pursuit of additional funding, both from the State and from outside grants. For Title I schools, increasing leverage to procure additional funding is an especially important goal because of the limited resources on which these schools tend to operate. Engagement of Title I schools – and increased reach to professionals in those schools with online training – thus remains a major focus for 2014–2019.

## Author Contributions

AN: conducted all data management and analysis, wrote the first draft of the manuscript, and implemented edits received from other authors. HC: assisted with data management and analysis and suggested edits to the draft manuscript. JD: worked with ALA to implement the intervention, supplied data to evaluators, and suggested edits to the draft manuscript. JF: assisted with implementing the intervention, supplied data to evaluator, and suggested edits to the draft manuscript.

## Conflict of Interest Statement

The authors declare that the research was conducted in the absence of any commercial or financial relationships that could be construed as a potential conflict of interest.
